# The Impact of Iterative Reconstruction on Computed Tomography Radiation Dosimetry: Evaluation in a Routine Clinical Setting

**DOI:** 10.1371/journal.pone.0138329

**Published:** 2015-09-18

**Authors:** Rachael E. Moorin, David A. J. Gibson, Rene K. Forsyth, Richard Fox

**Affiliations:** 1 School of Public Health, Curtin University, GPO Box U1987, Perth Western Australia, 6845, Australia; 2 School of Population Health, University of Western Australia, 35 Stirling Highway, Crawley, Perth Western Australia, 6009, Australia; 3 Department of Medical Imaging Science, Curtin University, GPO Box U1987, Perth, Western Australia, 6845, Australia; 4 School of Physics, University of Western Australia, 35 Stirling Highway, Crawley, Perth Western Australia, 6009, Australia; University of Texas at San Antonio, UNITED STATES

## Abstract

**Purpose:**

To evaluate the effect of introduction of iterative reconstruction as a mandated software upgrade on radiation dosimetry in routine clinical practice over a range of computed tomography examinations.

**Methods:**

Random samples of scanning data were extracted from a centralised Picture Archiving Communication System pertaining to 10 commonly performed computed tomography examination types undertaken at two hospitals in Western Australia, before and after the introduction of iterative reconstruction. Changes in the mean dose length product and effective dose were evaluated along with estimations of associated changes to annual cancer incidence.

**Results:**

We observed statistically significant reductions in the effective radiation dose for head computed tomography (22–27%) consistent with those reported in the literature. In contrast the reductions observed for non-contrast chest (37–47%); chest pulmonary embolism study (28%), chest/abdominal/pelvic study (16%) and thoracic spine (39%) computed tomography. Statistically significant reductions in radiation dose were not identified in angiographic computed tomography. Dose reductions translated to substantial lowering of the lifetime attributable risk, especially for younger females, and estimated numbers of incident cancers.

**Conclusion:**

Reduction of CT dose is a priority Iterative reconstruction algorithms have the potential to significantly assist with dose reduction across a range of protocols. However, this reduction in dose is achieved via reductions in image noise. Fully realising the potential dose reduction of iterative reconstruction requires the adjustment of image factors and forgoing the noise reduction potential of the iterative algorithm. Our study has demonstrated a reduction in radiation dose for some scanning protocols, but not to the extent experimental studies had previously shown or in all protocols expected, raising questions about the extent to which iterative reconstruction achieves dose reduction in real world clinical practice.

## Introduction

The relatively high doses received by patients from Computed Tomography (CT) were first highlighted in 1991 when estimates of organ and effective doses for common CT procedures on adults were reported in the UK.[1] These data provided the basis for reference doses published in 1999 as part of European guidelines on quality criteria for CT.[2] While it is recognised that advances in CT technology, such as the development of fast, helical scanning using multidetector rows / multislice have facilitated increasing clinical application of CT leading to significant improvements in health care for populations, concerns persist regarding the radiation dose to both populations and individuals from CT.

In the late 1990’s despite comprising only 2% of all medical imaging examinations, CT was shown to contribute a disproportionately large proportion (20%) of the collective population dose from diagnostic imaging.[3, 4] Surveys such as those conducted by the UK National Radiological Protection Board,[3, 5] also indicated that doses from CT were trending in a direction at odds with the decreasing collective patient doses received from conventional x-ray examinations.[3] During the 1990’s and early 2000’s CT examinations grew steadily to become a significant source of population exposure from medical radiology.[4] For example, in the UK, CT doubled both its contribution to the population effective dose (47%) and the proportion of all radiological examinations (4%) over a 10 year period.[6] Similar findings were reported in other developed countries such as Switzerland,[7] Germany,[8] the Netherlands [9] and the US.[10] In 2009 National Council on Radiation Protection and Measurements (NCRP) reported that CT and nuclear medicine contributed 36% of the total radiation exposure and 75% of the medical radiation exposure of the U.S. population.

As a result of these concerns the radiological community has made significant effort to reduce CT radiation dose via strategies such as tube-current modulation,[11] over-beam reduction,[12] organ-specific dose reduction (e.g. tilted gantry acquisitions)[13] and protocol optimization.[14] More recently an effort to reduce CT dose using iterative algorithms for image reconstruction has been pursued.[15–19] The algorithms have been shown to be capable of reducing image noise and thus in principle the radiation dose[16, 17] in the head,[17, 20, 21] chest,[22–28] abdomen[29–36] and CT angiography.[37–43] While these studies have shown the potential to significantly reduce radiation dose at the expense of forgoing a proportion of the noise reduction achievable using iterative reconstruction algorithms, the majority have been focussed on image optimization conducted in an experimental setting using either phantoms or very small samples of patients.

Since the amount of noise contained within the image is inversely proportional to the square root of the radiation dose[17] it follows that the potential for dose reduction using iterative reconstruction methods is directly related to the degree of noise acceptable to the radiologist. To date estimations of reductions in radiation dose achievable using iterative reconstruction methods have aimed to determine the maximum amount of noise that maintains acceptable diagnostic accuracy. Unfortunately due to the artificial/experimental circumstances of these studies the findings may not be representative of the degree of noise reduction acceptable in routine clinical practice. Thus the reduction in radiation dose indicated by these studies may not be realised or sustainable in a “real world” environment. Since any reduction in radiation dose mediated by iterative reconstruction requires negotiation and agreement regarding the associated changes in image quality by radiologists, a structured multidisciplinary implementation plan has been advocated.[44] The aim of this study was to evaluate the effect of introduction of an iterative reconstruction algorithm in the context of a mandated software upgrade on radiation dosimetry in routine clinical practice over a range of CT examinations.

## Methods

This was an observational study in the form of a natural experiment involving retrospective extraction of technical CT scanning data from a centralised Picture Archiving Communication System (PACS). Data were collected from scans undertaken at one secondary and one tertiary public hospital in Western Australia (WA) before and after the introduction of iterative reconstruction software (iDose, a hybrid iterative reconstruction algorithm) as a software upgrade initiated by the Department of Health level for all Philips Healthcare CT equipment in WA public hospitals. Upon introduction, iterative reconstruction was available for use as an alternative reconstruction algorithm. No systematic sustained implementation activities were undertaken to incorporate the new software into clinical practice. Both hospital sites had the same make and model 64 slice CT scanner. The secondary hospital had a single CT machine while the tertiary hospital had two identical machines. The study was approved by the Western Australian Department of Health Human Research Ethics Committee (#2011/97), with a waiver of informed consent for the retrospective review of electronic medical records.

### Data sources

Baseline (pre introduction of iterative reconstruction software) de-identified data were extracted manually from information contained in the dose report images for CT scans undertaken between 1^st^ January and 31^st^ May 2010 at both hospitals. Iterative reconstruction software was fully installed at both hospitals by the end of the first quarter of 2012 and data were extracted for the post installation phase from CT scans undertaken between September and November 2012 to allow sufficient time for use of the software to become imbedded into standard practice. For both pre and post software installation data capture periods a random sample of 20 adult cases from each of the CT examination types listed below (identified using the WA standardised CT coding system used in the PACS) provided data from both hospital unless indicated otherwise. Rarely fewer than twenty cases were identified within the collection period; in this event all cases were included in the study. If the technical parameters appeared particularly variable up to forty cases were collected. A sample of 20 cases is at least double studies using self-report data have used[6] is comparable in size to other studies[45], conforms to the European guidelines regarding the sample of cases required to assess dosimetry of usual practice and is consistent with the sample size used during estimation of Australian diagnostic reference levels [46, 47]) This random sampling method and sample size per protocol has also been used by the research team in previous studies [48, 49].The examination types included in the study were chosen because they represent approximately 70% of CT scans undertaken in WA.

1. Head non-contrast
2. Chest non-contrast
3. Chest pulmonary embolism (PE) study
4. CT angiogram thoracic aorta
5. CT coronary angiogram–*Tertiary hospital only*
6. Abdomen/Pelvis non-contrast
7. CT visceral angiogram—*Tertiary hospital only*
8. Chest/Abdo/Pelvis non-contrast—*Tertiary hospital only*
9. CT thoracic spine—*Tertiary hospital only*
10. CT lumbar spine

Protocol information (excluding the scout view) consisted of separate scanning sequences (phases) whenever present. The technical parameters collected included anatomical reference start-stop positions, volume weighted CT dose index (CTDIvol) and dose–length product (DLP).

### Radiation Dosimetry

Values of CTDIvol (inclusive of automated tube current modulation) and DLP were used to calculate the effective dose (mSv) for each sequence using the ImPACT dosimetry calculation software[50] as described previously.[48, 49, 51] The ImPACT dosimetry calculation software employs CT machine specific dosimetrics based on International Commission on Radiological Protection (ICRP) 103 tissue weighting factors[50, 52]. This tool allows organ and effective dose to be estimated in a population of patients based on Monte Carlo simulation in an idealized phantom. Justification of this method and its limitations are presented in the discussion section. Where appropriate cumulative protocol values of CTDIvol, DLP, organ dose and effective dose were calculated by summation of all sequences (phases) reported for each case.

### Effect of Iterative Reconstruction on CT Dose Metrics

The statistical significance of changes in the mean DLP and effective dose were evaluated separately for each CT examination type using the Mann-Whitney U test in each hospital setting and across both hospitals where CT examination types were undertaken by both hospitals. The Mann-Whitney U test is a rank-order test (or nonparametric test) for assessing the distribution of two independent groups when combined into a single sample (ie, whether the scores of two independent groups have a similar ranked distribution) not differences of means or medians. The test assesses the location and range of the lowest group's distribution within the overall sample range[53], and contrast this against a theoretical ranked distribution approaching normal ('U' or 'z' distribution, depending on sample size). The test is also powerful in detecting differences between group means and is commonly portrayed as the non-parametric substitute for Student's t-test when samples are not normally distributed[53]. This analysis was performed using SPSS version 19.

### Cancer Risk Modelling

In order to evaluate the impact on risk burden, of any statistically significant changes in effective dose, the age and Sex specific lifetime attributable risk (LAR) of cancer incidence pre and post installation of iterative reconstruction software was calculated in Microsoft Excel. This was achieved using the protocol specific organ dose and the age/sex-specific risk coefficients from table 12D-1 of the BEIR VII report[54]. The LAR of cancer incidence resulting from radiation dose to the remainder and ‘other’ organs was calculated using doses for organs included in the remainder organs by ICRP 103[52], and weighting them by the risk attributed by BEIR VII for ‘other’ organs. This method assumes all such organs contribute equally to risk which is clearly an approximation. The analysis was repeated for all combinations of age (ranging from 15 to 80 years) in yearly increments, using linear interpolation of the BEIR VII risk coefficients from the two nearest tabulated ages when data were not available for a specific age. The change in the LAR associated with the introduction of iterative reconstruction software was calculated as the difference between the two LAR estimates.

### Estimation of Potential Changes to Annual Cancer Incidence

Counts of the total number of CT examinations undertaken in Western Australia between 1^st^ January 2010 and 31^st^ December 2012 on adults aged 18+ years according to Sex and age for the examination types included were obtained from Medicare Australia (CT scan undertaken in the private sector) and the Western Australia PACS (CT scans undertaken in the public sector). For each CT examination scenario the average annual Sex/ age specific number of examinations was multiplied by the appropriate change in LAR observed in our study. The purpose of undertaking this analysis was to demonstrate that even small to modest reductions in radiation dose can have large impacts upon risk at the population level depending upon the Sex and age of the patients involved.

## Results

The mean, median, standard deviation, minimum and maximum values together with the minimum to maximum ratio for DLP and effective dose are reported in Tables 1 and 2 respectively for each examination type before and after the introduction of iterative reconstruction software according to hospital type. Absolute and relative changes in the mean DLP and effective dose differed according to CT examination and hospital.

**Table 1:** Mean, median and dispersion of dose length product (mGy.cm) according to CT scanning protocol before and after the introduction of iterative reconstruction (iDose).

	Before iDose software installation							Following iDose software installation						
Protocol Description	n	Mean	Median	St. Dev.	Min	Max	Min:Max Ratio	n	Mean	Median	St. Dev.	Min	Max	Min:Max Ratio
**Secondary Hospital**	** **	** **	** **	** **	** **	** **	** **	** **						
Head (Non-Contrast)	30	826.38	812.90	111.81	500.30	988.30	2.0	20	651.46	626.20	72.89	575.80	912.10	1.6
Chest (Non-Contrast)	20	433.18	412.95	162.20	161.80	664.90	4.1	20	282.88	248.60	119.42	62.10	473.20	7.6
Chest PE Study	21	501.92	315.40	520.84	159.80	2499.70	15.6	20	358.74	259.25	266.60	117.40	1157.60	9.9
Angiogram Thoracic Aorta	20	1304.01	1153.05	559.47	224.80	3017.00	13.4	20	1224.69	1247.95	490.69	510.40	2084.30	4.1
Angiogram Coronary	-	-	-	-	-	-	-	-	-	-	-	-	-	-
Abdomen +/- Pelvis (Non-Contrast)	20	568.10	531.50	309.17	156.70	1386.40	8.8	20	620.91	607.90	275.99	148.60	1187.00	8.0
Angiogram Visceral	-	-	-	-	-	-	-	20	-	-	-	-	-	-
Chest/Abdo/Pelvis (Non-Contrast)	-	-	-	-	-	-	-	-	-	-	-	-	-	-
Thoracic Spine	-	-	-	-	-	-	-	-	-	-	-	-	-	-
Lumbar Spine	20	620.66	592.40	276.00	296.10	1428.40	4.8	20	848.77	681.45	440.35	336.60	2088.40	6.2
**Tertiary Hospital**														
Head (Non-Contrast)	20	1207.76	898.55	647.32	842.30	2699.20	3.2	20	911.77	961.50	114.06	659.90	1055.60	1.6
Chest (Non-Contrast)	20	435.04	308.15	404.93	146.30	1872.90	12.8	19	224.33	176.60	176.27	28.30	583.40	20.6
Chest PE Study	20	738.23	747.45	329.85	41.80	1635.70	39.1	20	523.32	508.60	188.74	192.90	878.10	4.6
Angiogram Thoracic Aorta	20	799.86	830.20	352.21	192.40	1596.10	8.3	19	1029.48	1111.60	458.34	207.70	1938.80	9.3
Angiogram Coronary	20	747.35	355.75	691.26	141.30	2407.90	17.0	20	512.49	370.85	380.95	123.50	1682.40	13.6
Abdomen +/- Pelvis (Non-Contrast)	20	564.48	589.65	339.58	103.40	1169.10	11.3	19	593.59	530.90	344.28	110.70	1384.60	12.5
Angiogram Visceral	20	1865.1	1995.0	957.09	39.6	3599.8	90.9	20	2238.16	2025.30	1154.77	351.10	4731.80	13.5
Chest/Abdo/Pelvis (Non-Contrast)	10	997.21	832.40	415.11	463.40	1816.70	3.9	19	605.56	476.40	576.74	272.50	2921.80	10.7
Thoracic Spine	10	1032.26	1053.60	443.58	374.70	1787.90	4.8	19	629.91	479.40	527.08	158.20	1990.10	12.6
Lumbar Spine	10	990.89	831.85	601.24	432.90	2480.40	5.7	19	915.15	799.80	602.68	268.30	2852.50	10.6
**Both Hospitals**														
Head (Non-Contrast)	50	978.93	842.50	453.32	500.30	2699.20	5.4	40	781.61	701.90	162.17	575.80	1055.60	1.8
Chest (Non-Contrast)	40	434.11	346.30	304.47	146.30	1872.90	12.8	39	254.36	234.00	150.76	28.30	583.40	20.6
Chest PE Study	41	617.20	546.40	449.02	41.80	2499.70	59.8	40	441.03	380.95	242.75	117.40	1157.60	9.9
Angiogram Thoracic Aorta	40	1051.94	1049.20	527.35	192.40	3017.00	15.7	39	1129.59	1227.20	479.24	207.70	2084.30	10.0
Angiogram Coronary	-	-	-	-	-	-	-	-	-	-	-	-	-	-
Abdomen +/- Pelvis (Non-Contrast)	40	566.29	542.30	320.54	103.40	1386.40	13.4	39	607.25	571.20	308.29	110.70	1384.60	12.5
Angiogram Visceral	-	-	-	-	-	-	-	-	-	-	-	-	-	-
Chest/Abdo/Pelvis (Non-Contrast)	-	-	-	-	-	-	-	-	-	-	-	-	-	-
Thoracic Spine	-	-	-	-	-	-	-	-	-	-	-	-	-	-
Lumbar Spine	30	744.07	631.10	440.00	296.10	2480.40	8.4	39	881.11	753.30	519.75	268.30	2852.50	10.6

n = Number of cases included in analysis. Min = Minimum value, Max = Maximum value.

**Table 2:** Mean, median and dispersion of effective dose (mSv) according to CT scanning protocol before and after the introduction of iterative reconstruction (iDose).

	Before iDose software installation							Following iDose software installation						
Protocol Description	n	Mean	Median	St. Dev.	Min	Max	Min:Max Ratio	n	Mean	Median	St. Dev.	Min	Max	Min:Max Ratio
**Secondary Hospital**	** **	** **	** **	** **	** **	** **	** **	** **						
Head (Non-Contrast)	30	1.61	1.59	0.14	1.00	1.70	1.7	20	1.25	1.25	0.00	1.30	1.30	1.0
Chest (Non-Contrast)	20	8.57	8.00	3.43	2.96	13.97	4.7	20	5.44	4.85	2.14	2.29	9.27	4.0
Chest PE Study	21	9.88	6.43	10.10	0.48	48.70	101.9	20	7.07	5.19	4.89	2.38	22.44	9.4
Angiogram Thoracic Aorta	20	23.15	21.90	7.77	5.50	45.25	8.2	20	22.17	22.89	7.29	9.54	32.07	3.4
Angiogram Coronary	-	-	-	-	-	-	-	-	-	-	-	-	-	-
Abdomen +/- Pelvis (Non-Contrast)	20	9.07	8.53	4.33	3.74	19.81	5.3	20	10.29	9.38	4.46	4.08	18.90	4.6
Angiogram Visceral	-	-	-	-	-	-	-	-	-	-	-	-	-	-
Chest/Abdo/Pelvis (Non-Contrast)	-	-	-	-	-	-	-	-	-	-	-	-	-	-
Thoracic Spine	-	-	-	-	-	-	-	-	-	-	-	-	-	-
Lumbar Spine	20	11.98	11.08	4.15	5.98	23.05	3.9	20	14.33	10.99	7.13	6.31	33.42	5.3
**Tertiary Hospital**														
Head (Non-Contrast)	20	2.37	2.12	0.85	1.50	4.38	2.9	20	1.74	1.81	0.25	1.30	2.20	1.7
Chest (Non-Contrast)	20	7.93	5.81	8.21	2.54	41.09	16.2	19	4.21	3.51	3.53	0.57	12.22	21.5
Chest PE Study	20	14.60	14.94	6.62	0.83	32.46	39.0	20	10.48	10.61	3.46	3.49	17.50	5.0
Angiogram Thoracic Aorta	20	14.48	13.25	5.69	3.37	26.05	7.7	19	19.40	19.67	9.53	6.18	35.35	5.7
Angiogram Coronary	20	15.57	12.25	10.06	4.41	32.54	7.4	20	11.24	10.06	5.31	3.44	23.95	7.0
Abdomen +/- Pelvis (Non-Contrast)	20	9.69	9.71	5.41	2.06	19.76	9.6	19	9.96	9.53	5.59	2.00	26.11	13.1
Angiogram Visceral	20	31.17	31.05	15.22	0.63	59.20	93.7	20	37.19	33.07	15.95	5.63	64.38	11.4
Chest/Abdo/Pelvis (Non-Contrast)	10	17.34	14.63	6.55	9.26	31.04	3.4	19	14.53	7.93	27.04	4.77	125.66	26.3
Thoracic Spine	10	21.15	20.18	9.14	6.72	35.40	5.3	19	12.90	9.53	9.08	5.81	42.70	7.4
Lumbar Spine	10	15.02	14.68	4.13	10.08	22.80	2.3	19	16.09	13.01	9.74	5.24	43.94	8.4
**Both Hospitals**														
Head (Non-Contrast)	50	1.91	1.73	0.66	1.00	4.40	4.4	40	1.50	1.28	0.30	1.30	2.20	1.7
Chest (Non-Contrast)	40	8.25	6.73	6.22	2.54	41.09	16.2	39	4.84	4.34	2.93	0.57	12.22	21.5
Chest PE Study	41	12.18	10.65	8.81	0.48	48.70	101.9	40	8.77	8.54	4.52	2.38	22.44	9.4
Angiogram Thoracic Aorta	40	18.81	19.71	8.03	3.37	45.25	13.4	39	20.82	21.79	8.46	6.18	35.35	5.7
Angiogram Coronary	-	-	-	-	-	-	-	-	-	-	-	-	-	-
Abdomen +/- Pelvis (Non-Contrast)	40	9.38	8.87	4.84	2.06	19.81	9.6	39	10.13	9.53	5.00	2.00	26.11	13.1
Angiogram Visceral	-	-	-	-	-	-	-	-	-	-	-	-	-	-
Chest/Abdo/Pelvis (Non-Contrast)	-	-	-	-	-	-	-	-	-	-	-	-	-	-
Thoracic Spine	-	-	-	-	-	-	-	-	-	-	-	-	-	-
Lumbar Spine	30	12.99	11.56	4.33	5.98	23.05	3.9	39	15.19	11.81	8.44	5.24	43.94	8.4

n = Number of cases included in analysis. Min = Minimum value, Max = Maximum value.

Following introduction of iterative reconstruction software in the secondary hospital there were moderate reductions in mean DLP and effective dose in Head non-contrast, Chest non-contrast and Chest PE studies and very small reductions for Angiogram Thoracic Aorta. In contrast, an increase in both mean DLP and effective dose was observed for CT Lumbar spine and Abdomen/Pelvis non-contrast. Across all examination types, except lumbar spine, a reduction in the dispersion from the mean (standard deviation) for both metrics was observed.

In the tertiary hospital, reductions in the mean DLP and effective dose values were observed for Head non-contrast, Chest non-contrast, Chest PE study, Coronary Angiogram, Chest/Abdomen/Pelvis and Thoracic procedures. Increases in both dose metrics were observed for CT Angiography of the Thoracic Aorta, Abdomen/Pelvis CT and CT Angiogram Visceral. Changes in the dispersion of both metrics mirrored changes in the mean values i.e. when a reduction in the mean was observed a reduction in the standard deviation was also observed and vice versa.

When changes in radiation dosimetry were evaluated for CT examinations undertaken at both hospitals reductions in both the DLP and effective dose were observed for Head non contrast, Chest non-contrast and Chest PE study. However, increases in both metrics were observed for CT Angiography of the Thoracic Aorta, Abdomen/Pelvis CT and Lumbar spine CT. Changes in dispersion varied across the dose metrics with changes mirroring changes in the mean for effective dose; however DLP dispersion reduced in all examination types except CT of the Lumbar spine.

### Statistical Analysis of Changes in Dose Metrics

Table 3 shows the results of evaluating the statistical significance of the changes in the mean DLP and effective dose following introduction of iterative reconstruction software. In the secondary hospital statistically significant reductions in both the mean DLP and effective dose were only observed for Head non-contrast (-21% averaged over both metrics); and Chest non-contrast (-36% averaged over both metrics) examinations. In the tertiary hospital, while a statistically significant 27% reduction in the mean effective dose was observed in Head non-contrast CT examinations, the observed reduction in DLP of 24% was not statistically significant. In this setting statistically significant reductions in both metrics was observed for Chest (Non-Contrast) (-47% averaged over both metrics), Chest PE study (-28% averaged over both metrics), Chest/Abdo/Pelvis (Non-Contrast) (-27% averaged over both metrics) and CT of the Thoracic spine (-40% averaged over both metrics). With respect to examination undertaken at both hospitals two examination types showed statistically significant reductions in both DLP and effective dose, namely: Head non-contrast (-21% averaged over both metrics), Chest non-contrast (-41%) and Chest PE study (-28%).

**Table 3:** Evaluation of the effect of the introduction of iterative reconstruction (iDose) on dose length product (mGy.cm) and effective dose (mSv) across CT protocols.

	DLP (mGy/cm)^1^					Effective Dose (mSv)^2^					
	Difference in Means	% Difference in Means	Mean Rank^3^		Sig. (2-tailed)^4^	Difference in Means	% Difference in Means	Mean Rank^3^			Sig. (2-tailed)^4^
Protocol Description		(% of original if significant)	Before	After	p value		(% of original if significant)	Before		After	p value
**Secondary Hospital**											
Head (Non-Contrast)	-174.93	-21%	33.72	11.50	<0.001*	-0.36	-22%	34.83		11.50	<0.001*
Chest (Non-Contrast)	-150.30	-35%	25.65	15.35	0.005*	-3.14	-37%	25.60		15.40	0.006*
Chest PE Study	-143.18	-	23.48	18.40	0.175	-2.81	-	23.57		18.30	0.159
Angiogram Thoracic Aorta	-79.33	-	20.80	20.20	0.871	-0.97	-	19.90		21.10	0.745
Abdomen +/- Pelvis (Non-Contrast)	52.81	-	19.25	21.75	0.499	1.22	-	18.55		22.45	0.291
Lumbar Spine	228.11	-	17.25	23.75	0.079	2.35	-	19.20		21.80	0.482
**Tertiary Hospital**											
Head (Non-Contrast)	-296.00	-	19.85	21.15	0.725	-0.63	-27%		26.10	14.90	0.002*
Chest (Non-Contrast)	-210.70	-48%	24.10	15.68	0.021*	-3.72	-47%		24.55	15.21	0.011*
Chest PE Study	-214.91	-29%	25.15	15.85	0.012*	-4.12	-28%		25.10	15.90	0.013*
Angiogram Thoracic Aorta	229.62	-	17.00	23.16	0.092	4.92	-		17.35	22.79	0.136
Angiogram Coronary	-234.87	-	21.70	19.30	0.516	-4.33	-		22.30	18.70	0.330
Abdomen +/- Pelvis (Non-Contrast)	29.11	-	20.00	21.00	0.787	0.27	-		20.50	20.50	1.000
Angiogram Visceral	343.06	-	19.50	21.50	0.589	6.02	-		18.90	22.10	0.387
Chest/Abdo/Pelvis (Non-Contrast)	-391.65	-39%	22.40	11.11	0.001*	-2.81	-16%		22.50	11.05	0.001*
Thoracic Spine	-402.35	41%	20.20	12.26	0.017*	-8.25	-39%		20.50	12.11	0.012*
Lumbar Spine	-75.74	-	15.70	14.63	0.748	1.07	-		15.90	14.53	0.680
**Both Hospitals**											
Head (Non-Contrast)	-197.32	-20%	51.31	38.24	0.018*	-0.42	-22%		53.66	35.30	0.001*
Chest (Non-Contrast)	-179.75	-41%	48.68	31.10	0.001*	-3.41	-41%		48.93	30.85	<0.001*
Chest PE Study	-176.17	-	45.98	35.90	0.054	-3.41	-28%		46.24	35.63	0.042*
Angiogram Thoracic Aorta	77.65	-	37.28	42.79	0.285	2.01	-		36.53	43.56	0.173
Abdomen +/- Pelvis (Non-Contrast)	40.96	-	38.95	42.05	0.551	0.74	-		38.63	42.38	0.470
Lumbar Spine	137.03	-	31.60	37.62	0.217	2.19	-		34.13	35.67	0.753

^1^Dose length product, measured in mGy.cm

^2^Effective dose calculated using ImPACT in mSv

^3^Mean rank of DLP / Effective Dose before and following iDose. using the Mann Whitney U test (a rank-order (or nonparametric test) which assesses whether the before and after scores have a similar ranked distribution).

^4^Statistical significance of the difference in the mean rank DLP / mean Effective Dose following iDose compared with prior to iDose (ie probability of the scores belonging to a single group).

*Statistically significant (p<0.05) difference in mean rank DLP / Effective Dose following introduction of iDose.

### Impact on LAR by Statistically Significant Changes in Effective Dose

**Fig 1:**
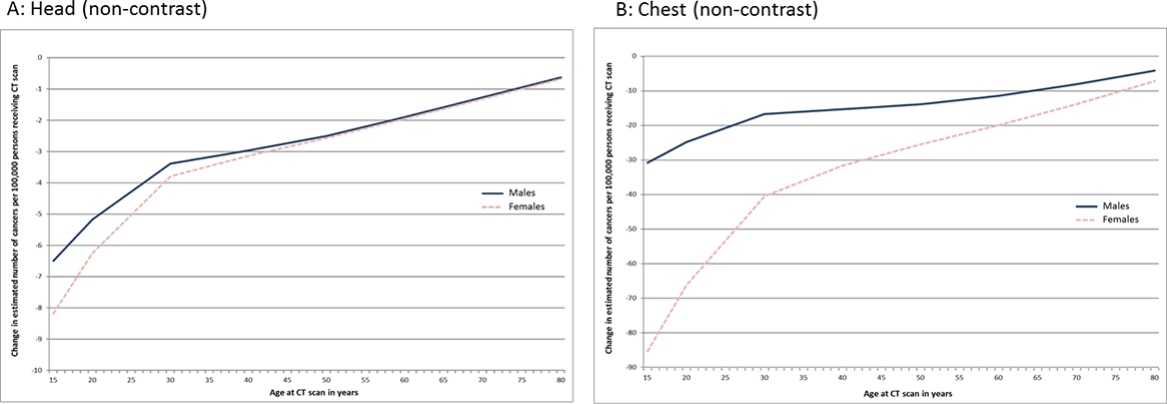
Change in the estimated number of incidence of cancers following installation of iterative reconstruction software (iDose) in a secondary hospital for CT protocols showing a significant change in mean effective dose.

Fig 1 shows the effect of the introduction of iterative reconstruction on risk of cancer for those examinations showing a significant difference in effective dose in the secondary hospital. The figure shows the effect on the estimated number of incident cancers per 100,000 examinations resulting from the change in effective dose attributed to introduction of iterative reconstruction according to Sex and age at the examination. It can be appreciated that for both examinations the significant reduction in the mean effective dose associated with the introduction of iterative reconstruction has translated to a reduction in the estimated number of incident cancers in both sexes (females to a greater extent than males) and that the magnitude of the reduction is greater at younger age groups. For CT head non-contrast the reduction in mean effective dose of 0.36mSv translated to 6.5 fewer incident cancers per 100,000 males and 8 fewer incident cancers per 100,000 females at age 15 years reducing to 0.7 per 100,000 regardless of Sex at age 80 years. For this examination type the reduction in the number of cancers is less than 1 per 100,000 for those exposed aged over 25 years. In comparison, for non-contrast CT of the chest the reduction in mean effective dose of 3.14mSv translated to 31 and 85 fewer incident cancers per 100,000 males and females respectively aged 15 years, reducing to 4 and 7 fewer incident cancers per 100,000 males and females respectively aged 80 years (at examination). The magnitude of the differential impact according to Sex reduces substantially after approximately age 30 years; however, it remains present across all ages in this examination type.

**Fig 2:**
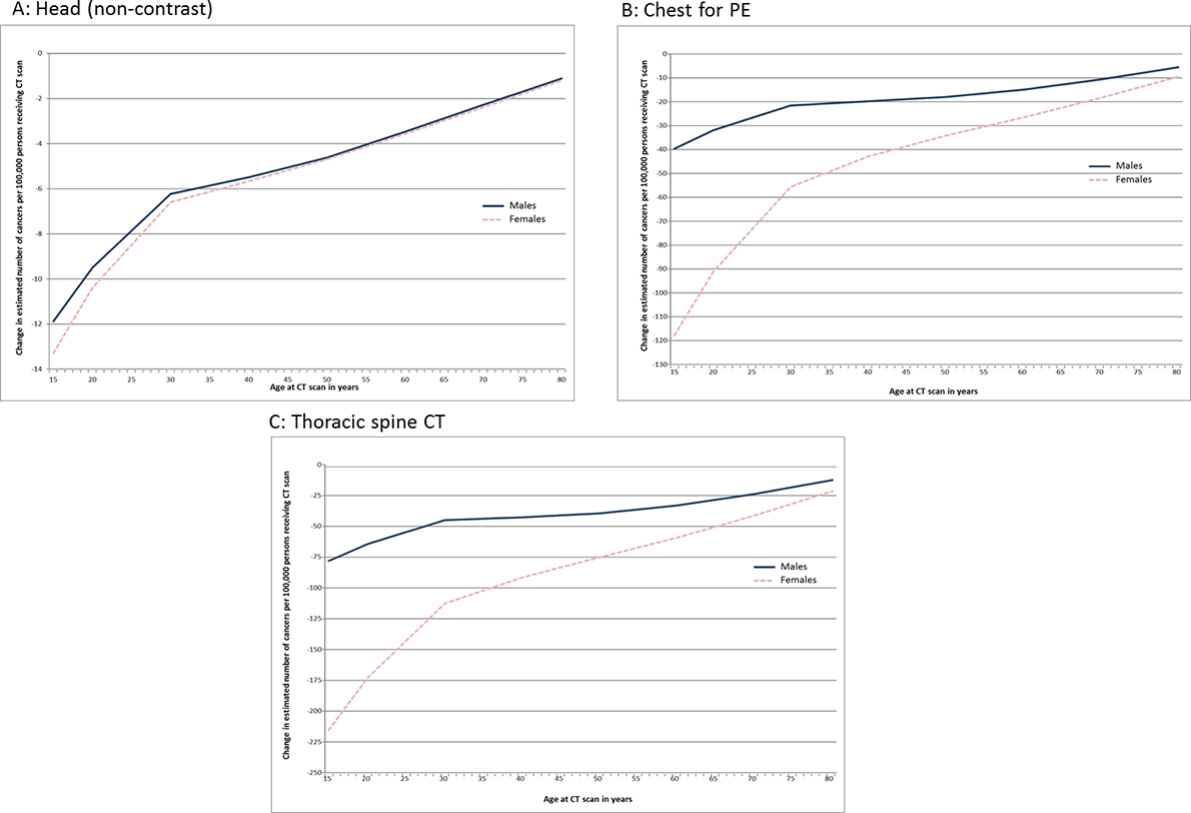
Change in the estimated number of incidence of cancers following installation of iterative reconstruction software (iDose) in a tertiary hospital for CT protocols showing a significant change in mean effective dose.

Fig 2 shows changes in risk for examinations having a significant difference in mean effective dose in the tertiary hospital. It can be seen that the reduction in the mean effective dose in head non-contrast examinations of 0.63mSv translated to an estimated reduction in the number of incident cancers of 12 and 13 per 100,000 males and females aged 15 years respectively, reducing to a reduction of approximately one per 100,000 males and females aged 80 years. The reduction in mean effective dose in Chest for PE study resulted in calculated reductions in the estimated incidence of cancer of 40 and 118 per 100,000 males and females aged 15 years, reducing to 5 and 10 per 100,000 males and females aged 80 years. The largest impact on cancer incidence was observed for CT of the thoracic spine where a reduction in the mean effective dose of 8.25mSv resulted in calculated reductions in the estimated incidence of cancer of 78 and 215 per 100,000 males and females aged 15 years, reducing to 12 and 21 per 100,000 males and females aged 80 years.

**Fig 3:**
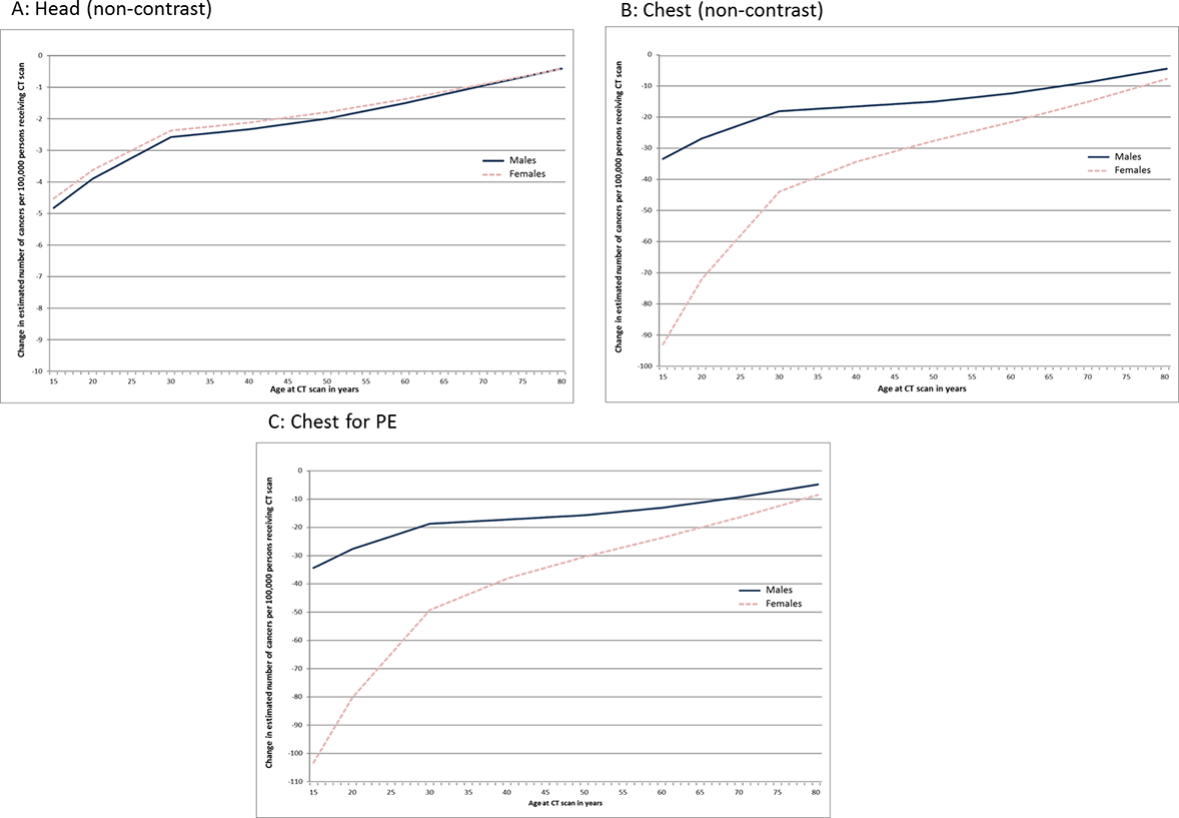
Change in the estimated number of incidence of cancers following installation of iterative reconstruction software (iDose) in both a secondary and tertiary hospital for CT protocols showing a significant change in mean effective dose.

Fig 3 shows the impact on cancer incidence for the three examinations that showed a statistically significant reduction in effective dose when undertaken at both hospitals. The reduction of 0.42mSv in effective dose translated to a reduction in the estimated incidence of cancer of 4.8 and 4.5 per 100,000 males and females at age 15 reducing to 0.4 regardless of Sex at age 80 years. Similar reductions in the number of estimated incident cancers were produced for the two chest examinations resulting from a reduction in mean effective dose of 3.41mSv with the small difference due to variation in the average scan length. At age 15 the estimated incidence of cancer reduced by 34 per 100,000 in males and 103 per 100,000 in females for the Chest PE study and 33 per 100,000 in males and 93 per 100,000 females for the Chest non-contrast study. At age 80 years the corresponding reductions were 5 and 8.5 per 100,000 males and females (Chest PE study) and 4.5 and 8 per 100,000 males and females (Chest PE non-contrast study).

### Iterative Reconstruction Effect on the Estimated Number of Annual Incident Cancers

Table 4 shows the reduction in the estimated number of annual incident cancers attributable to introduction of iterative reconstruction software in WA restricted to examinations where a significant reduction in effective dose was observed at both hospitals and Thoracic spine CT since this examination produced the largest effect on lifetime attributable risk. The results indicate that the impact of dose reduction depends on a combination of the magnitude of the reduction, the radio-sensitivity of the tissues in the scanning field, the age and sex of the patient and the volume of procedures undertaken. It can be seen that even though Head non contrast CT scans are the most frequently performed (59,888 examinations 2010–2012) due to the relatively small reduction in effective dose (-0.36mSv) and the relatively robust radio-sensitivity of tissues included in the scanning field the impact of introduction of iterative reconstruction was modest (0.71 fewer incident cancers annually). In contrast the number of Chest examinations (non-contrast and PE studies) is much lower but due to the greater reduction in effective dose (-3.41) and the relatively high radio-sensitivity of the organs the impact is greater (-2.81 incident cancers annually). Similarly although the largest reduction in effective dose (-8.25) and reduction in risk (Fig 3) was observed for CT of the Thoracic spine due to the relatively low number of examinations performed (2,547 between 2010–2012) the reduction in the estimated number of incident cancers annually is lower than for Head non-contrast (-0.66). While these annual reductions in cancers in WA may at first appear low, especially with respect to Head CT, when the change in cancer incidence rate per 100,000 is considered, the impact is substantial and falls within values considered moderate to high in epidemiology. In addition, the estimates produced in this analysis do not take into account the cumulative nature of the risk of ionising radiation.

**Table 4:** Potential impact on the annual incidence of cancer attributable to the introduction of iterative reconstruction software (iDose) in Western Australia.

			Number of CT examinations WA 2010–2012			
CT examination scenario^1^	Sex	Age at scan (years)	Public sector	Private sector	Change in cancer incidence rate per 100,000	Change in annual incidence of cancer in WA^2^
Head (Non-Contrast)–	Female	18–24	953	2,603	-3.5	-0.04
Both hospitals		25–44	4,154	10,654	-2.3	-0.11
		45–64	6,558	14,779	-1.6	-0.11
		65–84	9,565	14,267	-0.8	-0.06
		85+	6,579	2,642	-0.8	-0.02
	Male	18–24	1,759	1,886	-3.8	-0.05
		25–44	6,288	7,102	-2.5	-0.11
		45–64	8,358	10,841	-1.8	-0.12
		65–84	11,578	12,169	-0.8	-0.06
		85+	4,096	1,209	-0.8	-0.01
	**Total**		**59,888**	**78,152**	**-18.7**	**-0.71**
Chest examinations	Female	18–24	83	164	-64.1	-0.05
(non-contrast / PE study)-		25–44	599	2,070	-37.9	-0.34
Both hospitals		45–64	2,028	8,376	-23	-0.80
		65–84	2,798	9,269	-12.1	-0.49
		85+	414	864	-12.1	-0.05
	Male	18–24	117	203	-24.1	-0.03
		25–44	804	2,175	-16.6	-0.16
		45–64	2,792	8,688	-12.7	-0.49
		65–84	4,444	11,628	-7.1	-0.38
		85+	469	810	-7.1	-0.03
	**Total**		**14,548**	**44,247**	**-216.8**	**-2.81**
Thoracic Spine –	Female	18–24	34	40	-168.1	-0.04
Tertiary hospital		25–44	82	286	-106.4	-0.13
		45–64	127	590	-67.9	-0.16
		65–84	132	422	-36.1	-0.07
		85+	29	35	-36.1	-0.01
	Male	18–24	109	55	-62.8	-0.03
		25–44	312	360	-45.1	-0.10
		45–64	244	474	-36.1	-0.09
		65–84	151	271	-20.6	-0.03
		85+	20	14	-20.6	0.00
	**Total**		**1,240**	**2,547**	**-599.8**	**-0.66**

^1^Change in effective dose scenario under which the change in the number of incident cancers in the population is calculated.

^2^Based on the average annual number of examinations conducted in WA 2010–12 assuming all examinations are conducted under the scenario specified.

## Discussion

This study has utilised a large clinical data set, multiple CT scanning protocols across two hospitals to evaluate the magnitude of the change in radiation dosimetry (represented by DLP and effective dose) attributable to the introduction of iterative reconstruction software into routine clinical practice. It has also shown the potential effect these changes have in terms of risk burden by evaluating the lifetime attributable risk of cancer incidence, highlighting that even modest changes at the individual level can have large impact at the population level, particularly in younger women. Our findings indicate CT radiation dose can be reduced using interactive reconstruction techniques in day-to-day routine practice and these reductions can result in substantial lowering of the lifetime attributable risk. However, we found the magnitude of these reductions varied across anatomical site/protocol and was generally lower than indicated by experimental studies reported in the literature.

Previous experimental studies have shown reductions in radiation dose of between 20% and 30% are achievable in Head CT[20, 21, 55]; 30% to 95% in Chest CT[22, 24, 25, 28, 37, 56–59]; 40% to 65% in Abdominal CT[29–33] and 50% to 75% in CT Angiography[38, 40, 60] without compromising diagnostic accuracy. In our study we observed statistically significant reductions in the effective radiation dose for Head CT (22–27%) consistent with those reported in the literature. In contrast the reductions observed for Non-Contrast Chest CT (37–47%); Chest PE CT (28%) and Thoracic spine CT (39%) were towards the lower end of the range reported as achievable. The effective dose reduction observed in Chest/Abdo/Pelvis (Non-Contrast) CT (16%) was below expectations; in addition to no observed effective dose reduction for Abdomen +/- Pelvis (Non-Contrast) CT. Our study observed no reductions in radiation dose for CT angiography. This is discordant with findings from experimental studies that show large reductions are achievable for these examination types without loss of diagnostic accuracy.

We observed some discordance both across the two hospitals and across the metric used to evaluate radiation dose (DLP and effective dose). In terms of DLP, for Head CT we observed a statistically significant reduction of 21% in the secondary hospital but a non-significant reduction in the tertiary hospital. In contrast, in terms of effective dose we observed a statistically significant reduction for Head CT in both hospitals. A similar disparity between DLP and effective dose is observed in Chest PE study CT. The findings highlight that reporting radiation dose in terms of DLP, while easier to extract on a routine basis, is not the same as reporting it in terms of effective dose. While a significant change in effective dose but not DLP may seem intuitively contradictory it is important to understand the two measures are not 100% correlated. A DLP value can remain the same if the scan length is increased or decreased proportionally to any change in CT dose index volume (CTDI_vol_). However, DLP does not take in to account the radiosensitivity of the organs and tissues exposed. Effective dose combines the CTDI_vol_ with the radiosensitivities of organs and tissues within the scan field to generate dose estimates. It is possible changes in scan length, CTDI_vol_ can see a reduction in effective dose without a reduction in DLP by a narrowing of scan length to exclude radio sensitive organs outside areas of clinical interest. This is unlikely to be a direct result of changes to protocols to reduce dose using iterative reconstruction software. However, the introduction of iterative reconstruction may have triggered a broader review of standard protocol settings which contribute to effective dose.

The results for the two Chest CT protocols were also discordant across hospital sites. Statistically significant reductions in both DLP (35% in secondary hospital, 48% in tertiary hospital) and effective dose (37% in secondary hospital, 47% in tertiary hospital) were observed in both hospitals for Non Contrast Chest CT. However, Chest CT PE study showed statistically significant reductions of 29% for DLP and 28% for effective dose only in the tertiary hospital. When the data were pooled across hospital site for procedures conducted at both sites, statistically significant reductions were observed for Head CT and both Chest protocols (effective dose only for Chest PE study) evaluated due to improved power resulting from the doubling of the sample size. However no significant difference was observed for CT angiography, abdominal or lumbar spine studies when the data were pooled.

Our finding of mild to moderate reductions in radiation dose and no reduction in Abdominal or CT angiography suggests that while there is potential for substantial dose reduction using iterative reconstruction radiologists may not be willing to reduce the radiation dose thereby increasing the noise in some examinations in routine practice. The question posed by our results is: why doses remained the same, despite the use of iterative reconstruction that should have allowed doses to be significantly reduced, if the previous noise levels (i.e. prior to iterative reconstruction) had been acceptable? At least two explanations are possible (i) CT imaging factors were not modified following introduction of iterative reconstruction since existing protocols were retained without consideration of the potential for dose reduction or (ii) there was a conscious decision not to increase the image noise due to a belief that the reduced noise of iterative reconstruction provided greater diagnostic accuracy.

Non-acceptance of higher noise images than achievable (limiting the realisation of dose reduction) has been reported in the literature as an important challenge to the sustainability of dose-reductions predicted by experimental studies.[44] A system of multidisciplinary optimization of the radiation dose/image quality over an extended period of time following introduction of iterative reconstruction mayimprove acceptance of higher image noise levels and facilitate sustainable dose reduction.[44]

Nonetheless, the dose reductions observed in Head CT (both hospitals), Chest CT (both hospitals) and Thoracic Spine CT (tertiary hospital only) did produce notable reductions in estimated cancer incidence when applied to the WA state’s utilisation of these examinations and the wider population impact. While this analysis does assume an unrealistic level of conformity from the two hospitals included in the study to the entire state’s CT radiology providers, similar conformity has been assumed during discussions regarding dose reductions achieved by experimental studies to promote the effectiveness of iterative software., The purpose of our analysis was not to quantify ‘actual’ reduction in population dose, rather it was to demonstrate the population level impact of even small dose reductions. The cumulative efforts to reduce radiation dose from CT and other medical radiation exposures without sacrificing clinical validity has the potential to produce large cancer incidence rate changes at the population level.

### Study Strengths and Limitations

A limitation of our study is that our method of estimating effective and organ dose did not include size specific dose estimation (SSDE) methods since information regarding the body habitus of the patients included in the study were not available. While it is important to account for patient size when estimating individual patient radiation dose,[61] effective dose is intended to represent the dose to a population of patients (as we have done in our study) not individual patient dose.[62] This is an important distinction since effective dose is derived from measurements in an idealized phantom that integrates the relative weighting of the radiosensitive organs exposed and does not reflect the morphometrics of an individual patient.[62] All estimates of radiation dose have limitations, for example SSDE does not take into account variations in dose based on variations in scan length, assumes patients are centred in the CT gantry so that magnification effects are minimized and cannot be used for estimation of organ dose, and thus cannot be used to estimate effective dose.[62] Thus while SSDE is recommended and appropriate for estimating individual patient radiation dose it is not suitable when organ and effective dose estimates are required and is not necessary when estimating the average radiation dose characteristics of a particular examination i.e. examination specific rather than patient specific dosimetry is required.

This study was restricted to evluattion of a single manufacturer, machine model and type of iterative reconstruction. We acknowledge there are various forms of iterative reconstruction differently employed by individual manufacturers of CT machines and would likely produce different reduction results than observed in this study. This study did not seek to compare types of iterative reconstruction, rather it aimed to evaluate dose reduction in usual clinical practice (ie outside of an experimental setting) while controlling the variation between scanning protocols as best as possible by limiting the study to data from hospital departments which used the same type of CT machine (Philips Brilliance 64) and iterative reconstruction software.

A major strength of our study was the use of a random sample of CT patient data from an administrative data set containing CT scans undertaken during routine clinical practice. We aimed to estimate the average effective radiation dose for each adult CT protocol using a random sample of adult patients. The random sampling methodology was used to capture the underlying variation in doses produced for each scanning protocol at each time point and avoid recall or selection bias associated with the use of survey methods. The timing of the data collection was deliberately selected to allow for adequate time for each hospital to adjust and settle on revised protocols (if at all) after the introduction of iterative reconstruction. The use of actual scan parameters and dosimetry information recorded at the time of imaging rather than reliance on self-selected mean doses, ‘standard’ protocols or phantoms facilitate a more accurate representation of actual dose in practice (ensuring clinically accepted image quality was achieved since all examinations included were those conducted under a usual practice setting). Our data source and sampling method provide a more rigorous picture of real CT practice, rather than idealised or theoretical CT doses and practices obtained via experimental studies.

Detailed information regarding patient numbers according to CT protocols from routinely captured administrative data has allowed for cancer incidence attributable to CT scanning to be estimated. The estimates are extrapolations of the attributable cancer risk models developed in the BEIR VII report[54] using standard Monte Carlo methods modelling photon transport in CT. This study employs previously used methods to estimate risk and are the best available data[63]. While the BEIR VII report provides a framework for estimating age, sex and organ specific cancer risks from a radiation exposure it does not fully account for underlying pathology and life expectancy. The BEIR VII risks should be considered representative of the independent effect of radiation dose and can only be said to account for competing risks included in the original BEIR VII models. The estimated number of incident cancers is presented here as a demonstration of the magnitude of the effect on risk estimates attributed to the reduction in radiation dose associated with the introduction of iterative reconstruction. There is substantial difficulty in estimating population cancer risk as noted by the International Organization for Medical Physics (IOMP)[64]. In our study the imprecision is equally applied to both dose scenarios (pre and post introduction) hence the magnitude of the effect reported is not affected by the concerns of the IOMP. These concerns primarily rest with debate regarding acceptance of the ‘linear, no-threshold theory’ for ionising radiation exposure risk. The linear, no-threshold theory forms the foundation for radiation protection recommendations by international and national committees.[52, 54, 65] Criticism of the linear, no-threshold theory rest on statistical uncertainty for the relationship between radiation exposure and cancer incidence at low doses (less than 100mSv).[52, 66] However, current biological evidence does not support a threshold model where exposure to sub-100mSv radiation doses represents no risk.[54, 65, 67] Additionally, other studies estimating the cancer incidence resulting from the independent effects of CT radiation exposure have been published using the linear, no-threshold theory and BEIR-VII LAR estimates.[68, 69] Therefore our study has employed conservative, clinically representative, peer-reviewed and internationally recognised methodology for dose and risk estimation.

## Conclusion

Reduction of CT dose is a priority Iterative reconstruction algorithms have the potential to significantly assist with dose reduction across a range of protocols. However, this reduction in dose is achieved via reductions in image noise. Fully realising the potential dose reduction of iterative reconstruction requires the adjustment of image factors and forgoing the noise reduction potential of the iterative algorithm. Our study has demonstrated a reduction in radiation dose for some scanning protocols, but not to the extent experimental studies had previously shown or in all protocols expected, raising questions about the extent to which iterative reconstruction achieves dose reduction in real world clinical practice.

## Supporting Information

S1 Data De-Identified CT scanning machine settings and dose data by scan type, hospital and before or after dose modulation installation. (XLSX)

S2 Data Number of adult CT scans performed in Western Australia (2010 to 2012) by area scanned, sex, and age group. (XLSX)
